# Lack of basic rationale in epithelial-mesenchymal transition and its related concepts

**DOI:** 10.1186/s13578-024-01282-w

**Published:** 2024-08-20

**Authors:** Ying Cao

**Affiliations:** 1 The MOE Key Laboratory of Model Animals for Disease Study, Model Animal Research Center, Medical School of Nanjing University, 12 Xuefu Road, Pukou High-Tech Zone, Nanjing, 210061 China; 2 Jiangsu Key Laboratory of Molecular Medicine, Medical School of Nanjing University, Nanjing, China; 3 Shenzhen Research Institute of Nanjing University, Shenzhen, China

**Keywords:** Epithelial–mesenchymal transition, Epithelial state, Mesenchymal state, Neural stemness, Basic rationale

## Abstract

Epithelial–mesenchymal transition (EMT) is defined as a cellular process during which epithelial cells acquire mesenchymal phenotypes and behavior following the downregulation of epithelial features. EMT and its reversed process, the mesenchymal-epithelial transition (MET), and the special form of EMT, the endothelial-mesenchymal transition (EndMT), have been considered as mainstream concepts and general rules driving developmental and pathological processes, particularly cancer. However, discrepancies and disputes over EMT and EMT research have also grown over time. EMT is defined as transition between two cellular states, but it is unanimously agreed by EMT researchers that (1) neither the epithelial and mesenchymal states nor their regulatory networks have been clearly defined, (2) no EMT markers or factors can represent universally epithelial and mesenchymal states, and thus (3) EMT cannot be assessed on the basis of one or a few EMT markers. In contrast to definition and proposed roles of EMT, loss of epithelial feature does not cause mesenchymal phenotype, and EMT does not contribute to embryonic mesenchyme and neural crest formation, the key developmental events from which the EMT concept was derived. EMT and MET, represented by change in cell shapes or adhesiveness, or symbolized by EMT factors, are biased interpretation of the overall change in cellular property and regulatory networks during development and cancer progression. Moreover, EMT and MET are consequences rather than driving factors of developmental and pathological processes. The true meaning of EMT in some developmental and pathological processes, such as fibrosis, needs re-evaluation. EMT is believed to endow malignant features, such as migration, stemness, etc., to cancer cells. However, the core property of cancer (tumorigenic) cells is neural stemness, and the core EMT factors are components of the regulatory networks of neural stemness. Thus, EMT in cancer progression is misattribution of the roles of neural stemness to the unknown mesenchymal state. Similarly, neural crest EMT is misattribution of intrinsic property of neural crest cells to the unknown mesenchymal state. Lack of basic rationale in EMT and related concepts urges re-evaluation of their significance as general rules for understanding developmental and pathological processes, and re-evaluation of their significance in scientific research.

## Introduction

Since the initial description of the epithelial-mesenchymal transition (EMT) effect in the regulation of embryonic developmental process by Elizabeth Dexter “Betty” Hay (1927–2007, Harvard Medical School) [1], EMT has been reported as a universal cellular event involved in many different aspect of life process, including organogenesis, tissue repair, wound healing, inflammation, fibrosis, cancer progression, and even COVID-19 [2–10]. Particularly, EMT was employed to explain cancer metastasis initially but now has been implicated in every feature of cancer cells, including stemness, proliferation, evasion of death and immunosurveillance, dysregulated epigenetics, dysregulated metabolism, resistance to therapies, cancer heterogeneity, etc. [11–23]. EMT research has become a large research field and generated about 46,000 papers so far, and the number of publications is still growing rapidly. This makes EMT appearing as a mainstream concept [24]. Nevertheless, EMT research has been questioned since its beginning stage. It became flourishing afterwards, and discrepancies and inconsistencies of EMT effects in pathological processes, such as fibrosis, increase over time. In cancer, EMT was supposed to explain metastasis, but it failed to do so. EMT is a concept regarding the change from one cellular state to another, and the change is considered as a driving force during developmental and pathological processes. Unfortunately, after more than 50 years of EMT research, the epithelial and mesenchymal cellular states have not been clearly defined, and no markers have been identified as universal indicator of EMT. Also importantly, no evidence shows that loss of epithelial feature in epithelial cells could lead to a mesenchymal phenotype, and consequently, contributing to developmental or pathological processes. This means that on no basis EMT can be established as a scientifically meaningful concept. A concept without basic rationale cannot serve as a universal dogma dictating developmental biology and pathology.

## Outline of the history of EMT research

### Hay and the initiation of EMT research

It is generally credited that the American cellular and developmental biologist Elizabeth D. Hay played the pioneering role of EMT research [24, 25]. She observed initially that cartilage cells of limbs of *Ambystoma* larvae are able to dedifferentiate and re-differentiate again into cartilage cells, thereby contributing to limb regeneration [26]. Later, she found that regeneration of newt amputated limb needs the migration of epidermal cells over the wound surface of the limb [27]. These EMT-like processes implied that they may play important roles in wound healing and tissue regeneration, and led her to study the differentiation of epithelial cells and embryonic development. Hay and co-workers observed that extracellular matrix could influence differentiation of corneal epithelial cells [28]. Using chicken embryos as a model and optical and electronic microscopy, she could identify different cellular phenotypes in chicken embryos. At the 18th Hahnemann symposium in Baltimore, she reported how mesenchymal cells are transformed from epithelial cells during the migration of neural crest cells in neural tube formation. This was considered as a description of EMT effect before the term “EMT” was created. The 18th Hahnemann symposium was therefore considered as the birthplace of EMT research.

Around the 1970s, studies by other groups demonstrated epithelial-mesenchymal interactions during tissue formation and organogenesis, including heart, neural crest, Mullerian duct, intestinal brush border membrane, embryonic lungs, etc. [29–33]. In a publication reporting adult cells undergoing EMT in 1982, Hay and colleague used the term “epithelial-mesenchymal transformation” for the first time. They demonstrated that chicken lens epithelial cells cultured in vitro looked like mesenchymal cells and were able to move in collagen matrix [34]. Different terms or phrases were also devised by other groups to represent the EMT-like effect during the same period. Dulbecco and colleagues used “cuboid-to-fusiform transition” to describe their observation that cuboid epithelial cells of rat mammary tumors changed to fibroblast-like cells with fusiform morphology [35]. The phrase “rapid change from epithelial to mesenchymal character” was used by Illmensee’s group to represent an EMT-like effect observed during mouse embryogenesis [36].

In subsequent studies, Hay continued to describe the morphological changes during EMT, and tried to delineate EMT with molecular changes. For instances, her team showed that cultured embryonic lens epithelial cells underwent an EMT-like phenotypic change, and lose type IV collagen expression and γ-crystallin while expressing type I collagen (characteristic of mesenchymal cells) [37]. They also showed that thyroid epithelial cells undergoing EMT lose thyroglobulin but gain vimentin expression, suggestive of a dedifferentiation effect [38]. The term “epithelial-mesenchymal transition” appeared for the first time in a cited literature in a review by Hay and Zuk (1995) [39]. It became the official term after the first TEMTIA (The EMT International Association) meeting in 2003. “Epithelial-mesenchymal transition” was used instead of “epithelial-mesenchymal transformation” to distinguish it from the neoplastic transformation commonly used by cancer researchers [25].

### Transition from morphological to molecular description of EMT

After extensive phenotypic description of EMT, EMT research began to shift to molecular analysis. Hepatocyte growth factor (HGF) was observed to dissolve the junction proteins between epithelial cells, causing transformation of epithelial cells into migratory fibroblasts [40, 41]. Thiery’s group found that fibroblast growth factor 1 (FGF1) induced an EMT effect in rat bladder carcinoma cells, linking EMT to cancer [42]. Epidermal growth factor (EGF) was also shown to promote EMT in rat neonatal hepatocytes [43]. Transforming growth factor (TGF) family proteins were more extensively investigated for their roles in EMT. It was reported that TGF-α was able to induce a mesenchymal and invasive phenotype in rat prostate cancer cell [44]. TGF-β proteins were shown to play an important role in embryonic heart endothelial cells [45] and in embryonic palatal cells undergoing EMT [46], and mammary epithelial cells treated with TGF-β can undergo EMT [47]. TGF-β mediated EMT effect involves activation of TGF-β receptor and Smad signal transducers [48]. TGF-β receptor also activates Rho-GTPase, PI3K/AKT and MAPK pathways that can induce an EMT effect in embryonic chick heart, lens epithelial cells, renal epithelial cells, in vitro cultured tumor and non-tumor mammary epithelial cells [49–53]. In the pursuit of molecular mechanisms of EMT, Hay’s group demonstrated in 2008 that the Snail family of EMT activated transcription factors could induce TGF-β3 expression in cancer cell lines [54].

Studies of identifying molecular regulators of EMT increased dramatically in the 1990s. These led to the identification of EMT transcription factors (EMT-TFs), the first of which were Snail (Snai1) and Slug (Snai2) [55–57]. Nieto et al. (1994) showed for the first time that knockdown of Snail or Slug impaired EMT and subsequent cell migration during mesoderm and neural crest formation in chicken embryos [56]. Later, they were shown to promote an EMT effect in cancer cells [58–60]. In 2001, E12/E47 basic helix-loop-helix transcription factor (also called TCF3) was shown to evoke an EMT effect in MDCK kidney cells [61], and the ZEB family transcription factors, ZEB1 and ZEB2, were reported to induce an invasive phenotype in cancer cells [62], linking their function in regulating EMT. Later on, Weinberg’s group revealed that TWIST1 plays an essential role in cancer metastasis via promoting EMT [63]. These EMT-TFs were capable of inducing EMT-associated morphological and molecular changes, particularly transcriptional repression of the typical epithelial gene *E-cadherin* [58, 59, 61–66].

Jean Paul Thiery in the 1980s clutched “the gospel of developmental EMT to bravely jump from development to oncology, and finally grab cancer biologists by the scruff of the neck and force them to see the light. It was really from this point that the EMT field commenced its exponential growth” [24]. Since then, a large number of studies have demonstrated that EMT-TFs regulate not only cancer metastasis but eventually every aspect of cancer initiation and progression, and every feature of cancer cells, including stemness, unlimited cell proliferation, evasion of cell death and immunosuppression, chemoresistance, genomic instability, metabolic reprogramming, etc. Correspondingly, molecular mechanisms underlying regulation of cancer by EMT-TFs and regulation of EMT-TFs in cancer have also been extensively investigated [5, 11–18, 20–23, 67–70]. Due to their central role in EMT and extensive studies in cancer, ZEB1, ZEB2, SNAI1, SNAI2 and TWIST1 are considered as the core EMT-TFs [4, 17, 25]. Besides these core factors, a number of additional EMT-TFs have been identified, including FOXC2 [71], GSC [72], KLF8 [73], PRRX1 [74, 75], RUNX2 [76], SIX1 [77], TCF3 (also known as E47 or ITF1) [61], and TCF4 (also known as E2-2 or ITF2) [78].

## Mesenchymal-epithelial transition (MET) and endothelial-mesenchymal transition (EndMT)

Other two EMT-related cellular state transitions that have been extensively investigated are MET and EndMT. It is believed that transition from epithelial to mesenchymal state is reversible. The reversed process is known as mesenchymal-epithelial transition (MET) [19, 39, 79]. This means that mesenchyme derived from epithelium can sometimes revert back to the epithelial phenotype. At molecular level, MET is characterized by the decreased expression of mesenchymal factors and increased expression in epithelial markers, particularly E-cadherin [11, 19]. Putative roles of MET in embryogenesis and cancer have also been widely reported or proposed [11, 19, 39, 79].

The inner surface of all vessels in the body, including capillaries, arterioles, arteries, veins, and lymphatic vessels, is lined by a thin membrane-like structure, the endothelium. It plays primary roles in regulating and maintaining vessel wall permeability [80]. EndMT is cellular differentiation process by which resident endothelial cells delaminate and migrate away from the endothelium, progressively lose their endothelial features and acquire mesenchymal features. Accordingly, there is a tendency of decreased expression of endothelial markers and gain of mesenchymal marker expression in cells undergoing EndMT [80–83]. The molecular pathways regulating EndMT have been extensively investigated [84], which substantially overlap with those regulating EMT. Endothelial cells can be considered as a special type of epithelial cells. Therefore, EndMT is often considered as a special form of EMT. EndMT has been reported or proposed to play essential roles in many normal developmental and pathological processes, including cancer [80–85].

## EMT and development, fibrosis and cancer

It seems that EMT and MET are employed generally throughout embryogenesis to organogenesis. EMT was first observed during gastrulation in vertebrate embryos. Epithelial cells expressing E-cadherin and exhibiting apical-basal polarity in the epiblast layer (the definitive ectoderm) undergo EMT and move between the epiblast and hypoblast (the definitive endoderm) to form the third germ layer: the mesoderm [86–91], from which the embryonic and adult mesenchymal cells are derived. Conversely, MET turns early mesoderm into somites [92], which differentiate into dermamyotome, cartilage and bone by subsequent EMT [93]. This process of EMT is orchestrated by Wnt signaling pathway and requires the coordination of TGF-β and FGF pathways. Transcription factors Snail, Eomes, and Mesps also play important roles in regulating EMT during gastrulation [4]. It is believed that EMT drives the formation of migratory neural crest cells from neuroectoderm, leading to the loss of the original neuroepithelial morphology, dissociation from the neural folds, and gain of migratory phenotype with a fibroblast-like shape. Consequently, the cells disperse to the different parts of the embryo, where they undergo further differentiation. EMT associated with neural crest formation is also triggered by Wnt and FGF pathways and needs the activity of transcription factors such as Sox, Snail, Slug, and FoxD3 [4, 56, 94–98]. During organogenesis, EMT has been reported to involve in the formation of many different types of cells or tissues in an animal, such as fetal liver stroma [99], the cardiac cushion tissue [32, 100, 101], and oral palatal shelves [102–104].

It is believed that EMT also occurs as a repair-associated process during which the epithelial cells turn into fibroblast-like cells during tissue regeneration following traumatic and inflammatory damages. This process of EMT is associated with fibrosis or scarring in different organs, including liver, lung, kidney, and heart. During normal wound healing, myofibroblasts, which are mesenchymal cells, undergo apoptosis and disappear once upon the completion of re-epithelialization. Pathologically prolonged myofibroblast activity leads to fibrogenesis. In fact, persistent myofibroblast activation is a common feature of fibrogenesis, in which EMT is believed to play an essential role [8, 17, 25]. Myofibroblasts can be derived from a variety of sources. However, many lines of evidence showed that a major part of them are generated through EMT during organ fibrosis [8]. During kidney fibrosis, tubular epithelial cells turn into myofibroblasts via EMT and adopt fibroblast morphology, as evidenced by studies with animal models, human kidney biopsies, epithelial and mesenchymal marker staining, and lineage tracing with the mesenchymal marker FSP1 (also known as S100A4) [8, 105, 106]. It is believed that in lungs, epithelial cells experience repeated injury and persistent inflammation could undergo EMT, leading to fibrosis. The origin of myofibroblasts in lung fibrosis is not certain. Some studies showed that alveolar epithelial cells undergo EMT or partial EMT and contribute to fibrotic pathology. In a TGF-β1 murine model of lung fibrosis, β-galactosidase (β-gal)-labeling epithelial cells also expressed mesenchymal markers, indicating epithelial cells as the progenitors for the fibroblasts [8, 107–109]. Origin of activated myofibroblasts during liver fibrosis is also not clear, but epithelial cells undergoing EMT has been proposed as the source. Lineage-tracing studies with mouse models demonstrated hepatocytes underwent EMT, thereby contributing to the population of cells with the morphology of fibroblasts or expression mesenchymal markers [8, 110–112]. EMT regulated fibrogenesis following heart injury has been reported. Adult epicardial cells undergo EMT, and migrate into the injured myocardium where they generate different types of cells, including cardiac interstitial fibroblasts and coronary smooth muscle cells, to help tissue repair [8]. The role of EndMT during heart fibrosis has been more widely investigated because fibroblasts are derived from endothelial cells via EndMT [113–115]. During fibrogenesis of different organs, TGF-β signaling seems to play a general role in mediating EMT or EndMT.

Cancer has been the primary focus of EMT research. At the time of writing, 36,700 out of all ~ 46,000 EMT papers are studies dealing with cancer according to Pubmed. Among 4,246 EMT papers published in 2023, 3,325 are related with cancer. Since the initial studies about the link of EMT-TFs with cancer cell metastasis, EMT program mediated by EMT-TFs has been reported to endow nearly all malignant features to cancer cells, including stemness, fast cell cycle/proliferation, evasion of cell death and immunosuppression, therapy resistance, etc., and involve in nearly all aspects of carcinogenesis. Molecular mechanisms underlying how EMT-TFs regulates carcinogenesis or how EMT-TFs are regulated by other factors at gene, transcriptional and translational levels in cancer have been extensively reviewed [11–23, 68, 69, 116–118].

Based on EMT functions as described above, EMT is classified into three subtypes accordingly. Type I is associated with implantation, embryonic gastrulation and organogenesis during embryonic development, and does not provoke fibrosis or induce an invasive phenotype; type II plays roles in inflammation and fibrosis; and type III is involved in cancer [4].

## The controversies over EMT research on fibrosis and cancer

### The earliest arguments against EMT

Although EMT has become a formidable research discipline and a mainstream concept [24], it has been under intense debate since its early stage of study. When EMT events were increasingly reported during tissue formation and organogenesis around the 1970s [29–33], studies from two groups demonstrated the co-existence of differentiated and undifferentiated cell types, including epithelial and mesenchymal cells, in mesodermal mixed uterus tumors [119, 120]. Contrary to the view that mesenchymal cells are derived directly from epithelial cells, these studies considered that it was not possible for epithelial cells to acquire a mesenchymal shape or vice versa, and concluded that epithelial and mesenchymal cells share a common cancer stem cell origin [119, 120]. This viewpoint was not considered by mainstream research, of course. Nevertheless, it might reflect the truth (see text below).

### The controversies over EMT in fibrosis

With the progress of EMT research, the disputes over EMT have been also growing but primarily concentrated on the EMT effects in fibrosis of different organs. In a study in which double transgenic mice Alb-Cre × ROSA26-floxSTOPflox-LacZ were bred with transgenic mice expressing green fluorescent protein (GFP) driven by the collagen 1α1 promoter to generate triple transgenic mice in which β-galactosidase was expressed in “hepatocyte-derived” cells and GFP was expressed in “collagen-expressing” cells, transition of LacZ-positive (hepatocyte-derived) cells into GFP-positive (collagen-expressing) myofibroblasts in induced fibrotic liver was not detected [121]. By using Alfp-Cre × Rosa26-YFP mice in which the epithelial cells of the liver (hepatocytes, cholangiocytes, and their bipotential progenitors) are heritably labeled at high efficiency with yellow fluorescent protein (YFP), the study by Chu et al. (2011) showed that in induced liver fibrosis in Alfp-Cre × Rosa26-YFP mice, EMT did not occur because no evidence of colocalization of YFP with the mesenchymal markers S100A4, vimentin, α-SMA, or procollagen 1α2 was found [122]. Moreover, there was also no evidence for cholangiocyte EMT during hepatic fibrosis [122, 123]. These elaborate lineage-tracing studies argued against EMT in liver fibrosis. Thus, it was suggested that the term EMT should be abandoned in cholangiocyte biology [110–112, 124–127]. The roles of EMT in kidney and lung fibrosis are controversial, too. Cell fate tracing studies and absence of cells with mesenchymal morphology do not support EMT as an in vivo process in kidney and lung fibrosis [105–107, 112, 128–132]. Involvement of EndMT in fibroblast contribution during cardiac fibrosis is also not certain. Evidence of lineage-tracing studies showed that the majority of myofibroblasts after injury was derived from resident fibroblasts, but not from EndMT [115]. The reasons for the inconsistencies in the involvement of EMT or EndMT in organ fibrosis might be the unreliability fibroblast-specific protein-1 (FSP1/S100A4) as a mesechymal-specific marker to identify fibroblasts and cells undergoing EMT, and the unreliability of the detection of β-galactosidase colocalizing with FSP1 [112]. Absence of solid evidence raised the serious concern why EMT has become so deeply ingrained into fibrosis research [105].

### The disputes over EMT in cancer

As EMT was questioned intensely in fibrosis research, studies of EMT in cancer have been flourishing, and the number of papers had kept growing dramatically each year [25]. Nevertheless, some controversies over EMT in cancer also arose, including the two earliest arguments against EMT in mesodermal mixed uterus tumors [119, 120]. In 2005, Tarin pointed out that EMT is a misconception due to some reasons [133]. Firstly, it is difficult to define EMT precisely and most descriptions refer to changes in tumor cell morphology. Moreover, identification of cells as epithelial or mesenchymal based on shape and morphology or a few epithelial and mesenchymal markers is just subjective and unreliable. The earliest EMT effect is believed to occur during mesodermal formation in gastrulating embryos. However, the invaginating mesodermal cells in amphibian gastrulae are not spindle-shaped and do not lose cohesion with each other. Importantly, evidence of EMT in cancer metastasis is lacking [133]. Nevertheless, Tarin suggested that EMT in neural crest is of particular interests [133]. It was not surprising that EMT advocates did not agree with these points [134, 135]. One major piece of evidence supporting EMT in cancer is the downregulation of epithelial marker E-cadherin and upregulation of mesenchymal markers, particularly the core EMT-TFs, which predict invasiveness and metastatic potential and are negatively correlated with overall survival. Paradoxically, carcinoma cells within primary and metastatic lesions with well-differentiated epithelial morphology were also reported. Key epithelial markers, particularly E-cadherin, are expressed in invasive carcinomas [136], and E-cadherin is required for metastasis in multiple models of breast cancer [137]. The paradox is reconciled by MET in metastatic outgrowth, but the mechanism underlying activation of MET in metastatic cancer cells remains largely unknown [11, 138, 139]. It is a rather incomprehensible situation that both EMT and its reversed process contribute to metastasis. Despite these disputes, EMT studies in cancer grow and proliferate quickly, showing EMT as the endower of nearly all malignant features to cancer cells, as mentioned above. However, two studies in 2015, one using lineage tracing with Fsp1 or Vimentin promoter driving Cre recombinase (Fsp1-Cre or Vim-Cre) and the other using genetically engineered mouse models with deletion of Snail or Twist gene, demonstrated that EMT is not required for cancer metastasis but contributes to chemoresistance [67, 140]. Later, another two studies fought back by indicating that the markers used in the previous two studies are not universal markers for EMT programs or are not reliable as EMT markers [141, 142].

### Compromising the discrepancies in EMT studies

An appealing feature of EMT to cancer researchers is that EMT can convert adhesive and stationary state of epithelial cells into non-adhesive and individually migratory state of mesenchymal cells. Cell migration is fundamental for setting up and maintaining the correct organization of tissues/organs and body plan during animal development. In adults, cell migration is required for immune response, wound repair, and tissue homeostasis. Many cell types exhibit active migration, including collective migration of epithelial cells during gastrulation or lateral line primordium cells during development of fish, and single-cell migration of neural stem/progenitor cells during the development of the nervous system [143, 144]. Therefore, single-cell migration is not specific to mesenchymal cells, and epithelial cells are also not just stationary. Interestingly, mesenchymal cells also migrate collectively [145, 146]. This means that there is no clear-cut distinction in the migratory feature of epithelial and mesenchymal cells. The complex issue in migratory feature of epithelial and mesenchymal cells is compromised that EMT should not be interpreted as a binary switch from one cellular state to the other but should be interpreted as graded processes with a range of intermediate effects [146]. Meanwhile, tumor cells with co-expression of various epithelial and mesenchymal markers were frequently observed, meaning that transition from epithelial to mesenchymal state is a multi-step, multi-state, and dynamic process, ranging from a completely epithelial to a completely mesenchymal phenotype, as represented by the expression levels of epithelial and mesenchymal markers. Therefore, new terms ‘EMT-like’, ‘partial EMT’, ‘intermediate EMT’, ‘hybrid EMT’, or ‘dynamic EMT’, etc., were introduced [12, 17, 147–150], and the EMT concept itself was recommended to be elastic to compromise the discrepancies and complexity of EMT effect in cancer [25, 139, 150, 151]. Thus, ‘EMT plasticity (EMP)’ was suggested to replace EMT to reflect the high heterogeneity of EMT phenotypes [25, 68]. With the plasticity and elasticity, the EMT concept can now fit smoothly with any situation encountered in EMT research.

### Neural stemness representing the core property of cancer cells suggests that EMT in cancer represented by EMT-TFs is a misinterpretation

In 2017, co-workers and I reported that cancer cells are characteristic of neural stem cells or embryonic neural cells [152]. One reason is that inhibition of endogenous cancer promoting factors in cells of different cancer types led to neuronal-like differentiation in vitro, suggestive of the property of neural stem/embryonic neural cells, i.e., neural stemness. After comprehensive analysis on more than 3,000 cancer related genes, we found that most (if not all) cancer promoting genes or genes upregulated/activated in different cancer cells are neural stemness genes, or are specifically expressed or at least enriched in embryonic neural cells. By contrast, a major part of cancer suppressor genes or genes downregulated/silenced in cancer cells are non-neural genes in embryos. Therefore, cancer cells share the regulatory networks with neural stem/embryonic neural cells, thereby acquiring neural stemness in cancer cells [152]. In this study, it was noticed that core EMT-TF genes, which are upregulated in cancer cells and promote cancer, are embryonic neural genes, whereas the typical epithelial gene *E-cadherin*, a tumor suppressor gene [153, 154], is expressed in epidermis only, excluding embryonic neural tissues [152]. These patterns of EMT gene expression match very well with the rules about cancer promoting or suppressor genes mentioned above, and suggest that the EMT effects observed in cancer should be a misinterpretation. The EMT-TFs are a few components of neural regulatory networks that confer cancer cells with neural stemness, rather than mesenchymal state. In-depth analysis revealed that, unfortunately, the so-called epithelial and mesenchymal states in the EMT concept have remained unclear or undefined in spite of large scales of EMT research. In combination with other studies in cancer and developmental biology, I proposed that cancer initiation and progression represent a process of progressive loss of original cell identity and gain of neural stemness. Meanwhile, the plausibility of EMT concept itself, but not merely its roles in cancer, was put into question because what are the general epithelial and mesenchymal states is still unknown [155]. In 2020, after two years of discussion, TEMTIA published a consensus statement about the guidelines and definitions for EMT research due to discrepancies in data interpretation and persistent disagreements about whether the process studied is EMT [25]. The consensus statement listed some critical problems about EMT and EMT research. Firstly, “while the characteristics of fully epithelial cells are relatively clearly defined, our current knowledge does not allow us to define the mesenchymal state with specific cellular characteristic or molecular markers that are universal end-products of all EMT programmes”, indicating that the epithelial state is relatively known but the mesenchymal state is unknown. Most EMT studies have been concentrated on a few EMT factors/markers. However, “EMT status cannot be assessed on the basis of one or a small number of molecular markers”. Therefore, “the primary criteria for defining EMT status should be changes in cellular properties together with a set of molecular markers, rather than relying solely on molecular markers” [25].

Subsequent studies of mine revealed that neural stemness is the key cellular property determining and unifying tumorigenicity and pluripotency, which govern tumorigenesis and embryogenesis, respectively. Such a superiority of neural stemness is predestined by the evolutionary advantage of neural genes and neural cell state [156–161]. Characterization of neural stemness and its regulatory networks revealed that they determine malignant features and tumorigenicity of cancer cells. It is hard to know what the undefined mesenchymal state shares in common with cancer cells [156, 157].

## Reassessing the basic rationale of EMT concept

Analysis above indicates many discrepancies and defects in EMT research, it also casts doubts on the plausibility of the EMT concept.

### The epithelial and mesenchymal states have not been defined

According to the consensus statement on the guidelines and definitions for research on epithelial–mesenchymal transition by TEMTIA, “Epithelial–mesenchymal transition (EMT) is a cellular process during which epithelial cells acquire mesenchymal phenotypes and behavior following the downregulation of epithelial features” [25]. This means that the plausibility of EMT concept depends entirely on the understanding of the phenotypes and behaviors of epithelial and mesenchymal cells. In fact, epithelial and mesenchymal cells are highly heterogeneous populations of cells with diverse phenotypes and functions. In general, epithelial cells are tightly packed together in cell sheets, form covering on all internal and external surfaces of animal body, and make up lining of hollow organs. During early embryogenesis, pluripotent epiblast cells are considered as the earliest epithelial cells. Later, there are epithelial cells, including neuroepithelial cells, that give rise to neural crest cells and palatal epithelial cells, etc. Types of epithelial cells are more diverse in adults, since each organ is covered by epithelial cells specific to the type of organ, such as those in skin, lung, kidney, etc. This means that epithelial cells of different tissues/organs have different intrinsic regulatory networks to define cell properties including tissue or organ-specific functions. For example, epithelial cells of lung, which is derived from endoderm, must be different in function and cellular property and regulatory networks from those of kidney or skin, which are derived from mesoderm and ectoderm, respectively. During embryogenesis, mesenchymal cells are derived from mesoderm and form multipotential embryonic connective tissue, and give rise to all adult connective tissues, as well as the lymphatic and circulatory systems. In adulthood, mesenchymal cells are commonly described as non-epithelial, non-hematopoietic and non-endothelial cells that support and connect tissues, including muscle, tendon, and fat tissues, and encompass diverse populations of fibroblasts, stromal cells, pericytes, perivascular smooth muscle cells and mesenchymal progenitors.

Heterogeneity of the types of epithelial and mesenchymal cells raises the question whether there exist the general states or properties of all epithelial cells and mesenchymal cells based on which EMT can be established as a scientifically meaningful concept and serves as a general rule to explain developmental and pathological effects. Stable epithelial cell–cell junctions, apical–basal polarity and interactions with basement membrane are recognized as the common features of epithelial state [25]. However, these are just an integral part of the property of a particular type of epithelial cells. For example, when epiblast cells turn into embryonic mesenchyme during gastrulation, not only do they lose their apical–basal polarity, change their cytoskeleton and show decreased cell–cell adhesion, but their regulatory networks defining epiblast pluripotency are also changed overall to the networks defining non-pluripotent mesodermal cells. During carcinogenesis of the lung, epithelial cells lose not only cell adhesion, but also their function in respiration. Correspondingly, the regulatory networks change in addition to the decreased expression of epithelial markers, e.g., E-cadherin. Moreover, epithelial cells show a wide range of differentiation potential, from pluripotent epiblast cells to terminally differentiated epithelial cells in different organs. Therefore, focus on the loss of epithelial state only in the EMT concept is an oversimplification and biased interpretation of the change in cellular properties and regulatory networks of epithelial cells. The mesenchymal state is more confusing, because there has been no way to define this cellular state with specific cellular characteristics or molecular markers [25, 155, 157]. Therefore, EMT means a transition from an almost unknown cellular state to an unknown cellular state. It is incomprehensible how an unknown cellular state can be used as standard reference for the properties of other cells or endows different cellular properties to cancer cells, and how EMT can be a scientifically meaningful concept. No matter whether EMT is interpreted as a binary switch from one cellular state to the other or as graded processes with a range of different outcomes, ‘EMT-like’, ‘partial EMT’, ‘intermediate EMT’, ‘hybrid EMT’, and ‘dynamic EMT’, and ‘EMT plasticity’, express no essential difference from EMT because they all depend on the understanding of mesenchymal state. Classification of the three subtypes of EMT is superfluous when what is EMT is unknown. Similarly, MET and EndMT are also groundless concepts without knowing the mesenchymal state. It was claimed that a pressing issue for EMT is to resolve the controversy on the contribution played by EMT in metastasis [24, 139]. A more pressing issue to resolve seems to be whether EMT is a plausible concept.

### EMT as a secondary but not causal effect during cell state transition

EMT is considered as a general rule that drives developmental and pathological processes. However, it has not been confirmed whether the change from epithelial state to mesenchymal state observed in vivo is a cause, consequence or just an accompanying event of the change in overall cellular property during developmental or pathological processes. The EMT community considers that mesoderm and neural crest formation are typical events driven by EMT. Nevertheless, it is well characterized that mesoderm formation is induced by signals from hypoblast or endoderm [86, 162], and neural crest formation is induced by interaction of neural plate with adjacent non-neural cells [163–167]. Therefore, loss of epithelial state and gain of mesenchymal state should be a subsequent but not causal effect. Similarly, the loss of epithelial state and gain of mesenchymal state during carcinogenesis and other pathological processes might be also a secondary effect caused by signaling cascades driven initially by different factors, e.g., cancer-driving mutations in *KRAS*, *TP53*, etc. E-cadherin is a key adhesion molecule, and its loss is considered as the hallmark of EMT [138]. It is funny that E-cadherin loss does not cause an EMT effect [168]. *E-cadherin* knockout causes defects in embryos and organs, and promotes tumorigenesis. However, no EMT effects were observed in the defects or tumorigenesis in response to E-cadherin loss [169–172]. EMT is characteristic of cytoskeleton change. Cytokeratins are intermediate filament proteins predominantly used for cytoskeleton formation in epithelial cells, whereas mesenchymal cells use Vimentin. It would be expected that cytokeratin should inhibit EMT. By contrast, experiments showed that it promotes EMT [173, 174]. This means that loss of epithelial feature alone cannot lead to mesenchymal feature in cells. In vitro studies showing EMT effects induced by growth factors and EMT-TFs [40–42, 47, 62, 63], also mean that loss of epithelial feature and gain of mesenchymal feature in cells is a secondary but not a driving effect. It is a commonsense that isolated epithelial cells cultured in vitro cannot acquire mesenchymal feature without the presence of inducing factors. Interestingly, the secondary effect was progressively interpreted as a driving factor contributing to developmental and pathological processes with progression of EMT research. In fact, latest studies confirmed that mesoderm and neural crest formation is not driven by EMT [175, 176].

### Interpretation of EMT and the functions of EMT-TFs in the context of embryonic development

It remains an essential question how to interpret the ‘EMT’ effects. In the context of developmental biology, embryonic development is a progressive process of differentiation from the totipotent unicellular state of a fertilized egg to the pluripotent state of inner cell mass and epiblast cells, which further differentiate into multipotent/oligopotent/unipotent progenitor/precursor cells of tissues/organs of different lineages. These cells finally differentiate into different types of mature and functional cells of tissues/organs. In the context of EMT/MET, however, the progressive differentiation of embryonic cells and the change in cellular properties and corresponding regulatory networks are described only as transitions between the undefined epithelial and mesenchymal states. This is confusing for understanding embryogenesis. The renowned example of EMT, in which epiblast cells turn into embryonic mesenchymal cells, is the differentiation of epiblast cells into mesodermal cells induced by signals from hypoblast cells. The commonality of mesoderm induction in different vertebrate embryos has been extensively investigated [162]. EMT is not suggested to play a role in mesoderm induction. Neuroepithelial cells turning into migratory neural crest cells is another typical EMT event. According to definition, mesenchymal cells are cells from mesodermal lineage. However, neural crest cells are precursors of the peripheral nervous system, which belongs to the neural lineage, and the general epithelial marker E-cadherin is not expressed in neuroepithelium [152, 177]. Neuroepithelial and neural crest cells are of particular interest, which will be discussed later. The true meaning of the ‘EMT’ effects observed during organogenesis and fibrosis based on marker expression or lineage-tracing studies is unclear and needs re-evaluation.

**Fig. 1:**
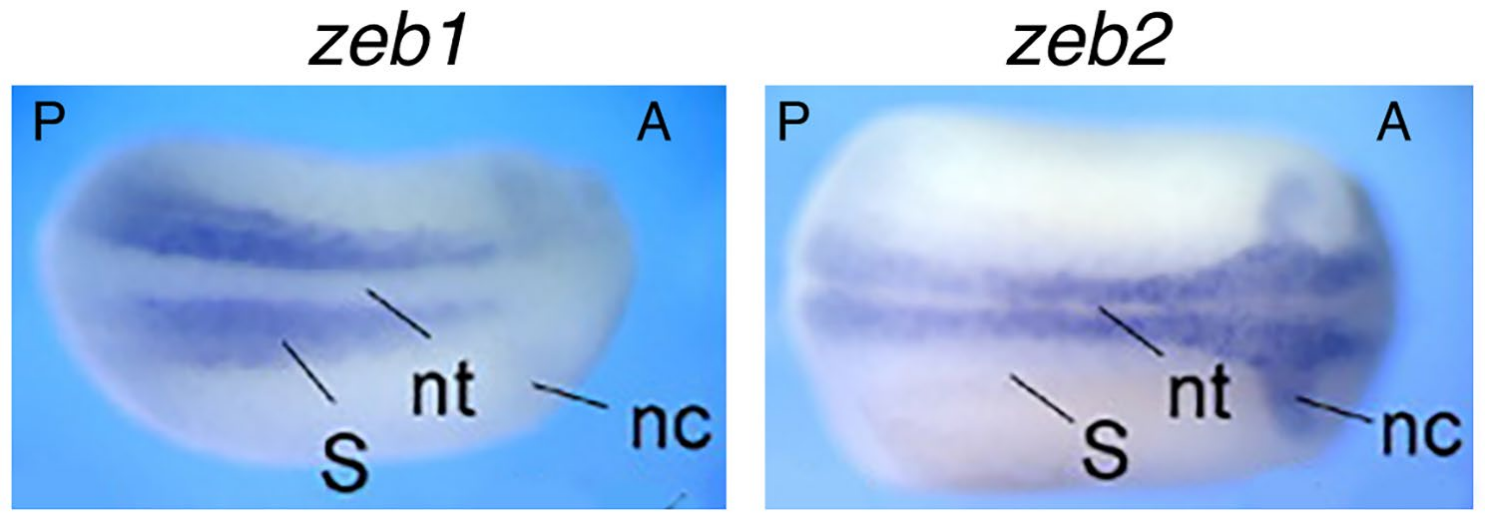
Distinct expression patterns of *zeb1* and *zeb2* in neurula embryos of *Xenopus*. Whole mount in situ hybridization revealed specific expression of *zeb1* in paraxial mesoderm (somites) excluding embryonic neural tissues, whereas *zeb2* is localized to neural plate and neural crest, the precursor tissues of the central nervous system and peripheral nervous system, respectively. Dorsal view is shown for each embryo with the anterior (A) to the right. A, anterior; nc, neural crest; np, neural plate; nt, neural tube; P, posterior; S, somites. Expression pattern data were from van Grunsven et al. (2006) [183] with permission from publisher

**Fig. 2:**
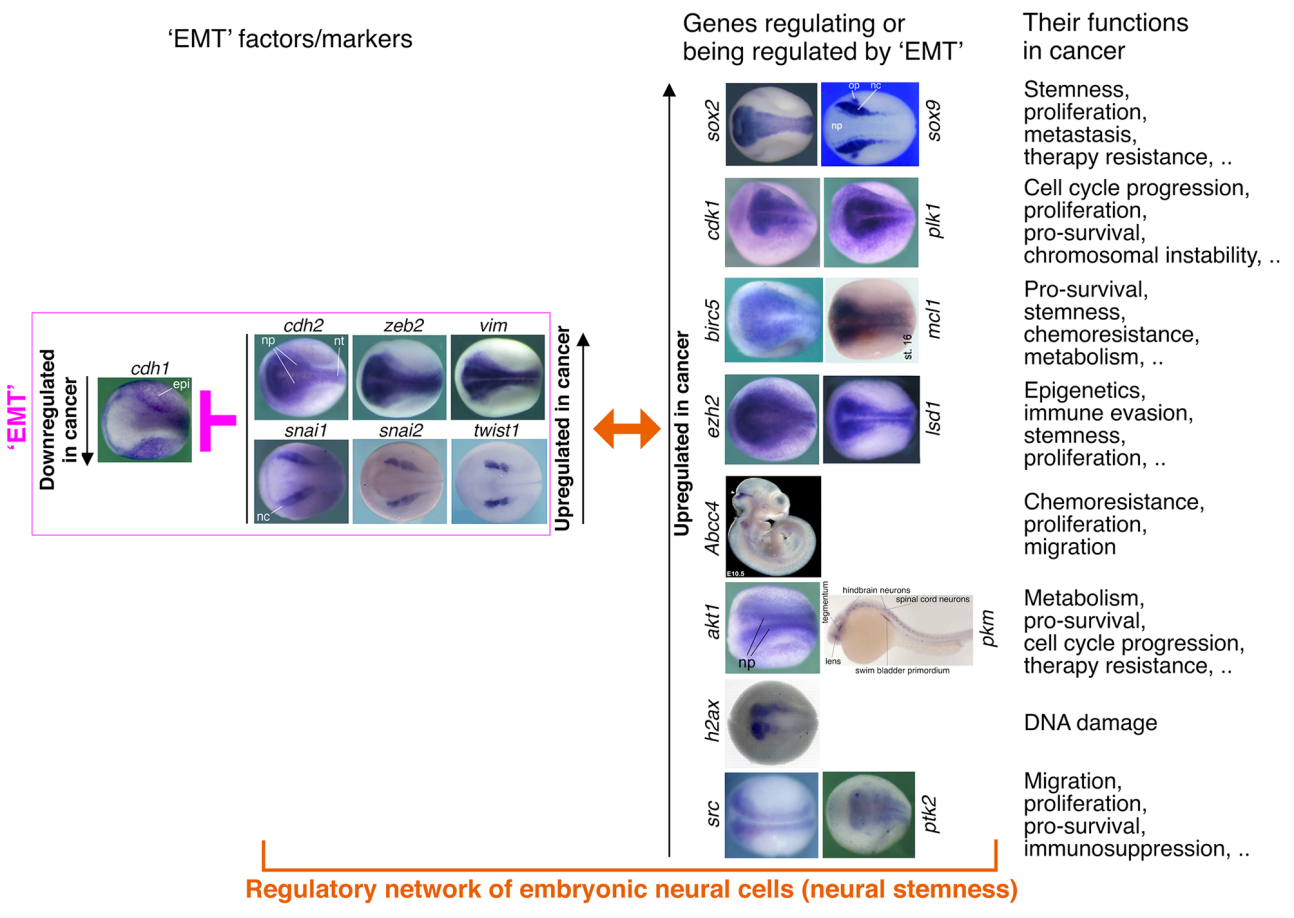
Localized embryonic expression of ‘EMT’ factors/markers and the genes regulating or being regulated by ‘EMT’ in cancer reveals that they are components of the regulatory networks of embryonic neural cells, suggesting the key role of neural stemness rather than the unknown mesenchymal state in the determination of different features of cancer cells. Epidermal-specific expression of the typical epithelial gene *cdh1* (*E-cadherin*) and the neural expression of ‘EMT’ factor/marker genes and other genes suggest strongly their regulatory relationship, which was demonstrated in many studies (see text). Expression patterns were detected with whole mount in situ hybridization. Expression pattern of *Abcc4* in mouse embryo was from Jukkola et al. (2006) [242]. Expression of *pkm* (*pkm2*) in zebrafish embryo was from Thisse et al. (2001) [243]. Expression of other genes was detected in *Xenopus* embryos. Expression patterns of *cdh1*, *cdh2*, *vim*, *snai1*, *sox2*, *cdk1*, *plk1*, *birc5*, *ezh2*, *lsd1*, *akt1* and *ptk2* were from Zhang et al. (2017) [152]; *sox9* was from Lee and Saint-Jeannet (2011) [244]; *mcl1* from Sena et al. (2020) [245]; *h2ax* from Lee et al. (2010) [246]; *src* from Lewis et al. (2017) [247]; and *snai2* and *twist1* were from Wang et al. (2015) [248]. Reuse of expression pattern data was permitted by publishers. Dorsal view was shown for *Xenopus* embryos, with the anterior of embryos to the left. epi, epidermis; nc, neural crest; np, neural plate; nt, neural tube

Core EMT-TFs have been extensively studied for their roles in EMT. Nevertheless, numerous other studies revealed their functions beyond EMT. Zeb2 is critical for exit from the epiblast state in mouse ESCs and for neural and general differentiation [178]. Mice with homozygous mutation of *Zeb2* display defects in neural tube closure, early arrest of neural crest cell migration, and absence of neural crest cells. Meanwhile, *E-cadherin* expression domain extends to the neuroepithelium in mutant mice. By contrast, homozygous *Zeb1*-deficient mice exhibit multiple skeletal defects but no distinctive phenotypic change in the central nervous system [179]. Zeb1 and Zeb2 exhibit opposite functions in *Xenopus* embryos. Overexpression of Zeb2 led to neutralization/dorsalization of embryos with extra formation of neuroectoderm and decreased epidermal ectoderm, and overexpression of Zeb1 induced ectopic formation of mesoderm without change in neuroectoderm [180]. Latest studies showed that ZEB1 is required for the mesodermal-to-myogenic specification but ZEB2 promotes neural fate specification of human embryonic stem cells. Moreover, ZEB1 functions as an inhibitor rather than an inducer of EMT [181, 182]. It can be seen that ZEB2 is mainly involved in regulation of neural development, while ZEB1 is principally in mesodermal tissue differentiation. The functional difference corresponds to their expression patterns during embryogenesis. *zeb1* expression is localized to the paraxial mesoderm, which gives rise to somites, the precursor of muscle and skeleton; whereas *zeb2* is selectively expressed in the precursor tissues of the nervous system during embryogenesis, including neural plate and neural crest [183] (Fig. 1). Similar expression patterns of *Zeb1* and *Zeb2* are also present during mouse embryonic development [179]. TWIST1 and its orthologues are involved in regulation of gastrulation and body axis patterning of *Drosophila* embryos [184], pluripotency and differentiation of embryonic stem cells [185], mesoderm differentiation, differentiation of embryonic hematopoietic stem/progenitor cells [186], and particularly, cell fate decision of neural crest and development of neural crest derived structures [187–190]. Snai1 and Snai2 were intensely studied in the specification and migration of neural crest in vertebrates [56, 191–193]. Similar to ZEB2, SNAI1, SNAI2 and TWIST1 are mainly involved in regulation of neural development. Correspondingly, expression of *snai1*, *snai2* and *twist1* is localized or at least enriched in neural plate and neural crest at the neurodevelopmental stage [152, 157] (Fig. 2). Therefore, the contrasting roles of ZEB1 and ZEB2, together with the functions of other EMT-TFs, match exactly with their localized expression patterns that reflect their endogenous functions in different tissue differentiation or specification during embryonic development but not EMT. These functional studies demonstrated that the EMT-TFs are simply developmental factors. A key piece of evidence for these proteins functioning as EMT-TFs is their repression of *E-cadherin* transcription. Such a regulatory relationship is also reflected by that *E-cadherin* is specifically expressed in epidermis, excluding from the expression domains of EMT-TF genes (Figs. 1 and 2).

EMT-TFs are generally upregulated or activated in cancer cells and promote cancer progression. By contrast, E-cadherin is generally downregulated in cancer cells and functions as a cancer suppressor. This fashion of expression change in EMT genes is actually within a much broader range of gene expression change in cancer cells. Detailed investigations on cancer genes and the basic property of cancer cells suggest that it is neural stemness, but not the unfathomable mesenchymal state, that is the endower of not only malignant features and tumorigenicity but also pluripotent differentiation potential to cancer cells [156, 160, 161].

### EMT and EMT-TFs in cancer: misattribution of the role of neural stemness to mesenchymal state

EMT, which is symbolized by EMT factors, is believed to be a driving force for cancer progression. However, neural specific or enriched expression of EMT-TFs during embryogenesis implies otherwise. It was generalized that most cancer promoting genes, including those for EMT-TFs, are neural stemness genes or genes with specific or at least enriched expression in embryonic neural cells, and the embryonic neural regulatory networks confer neural stemness to cancer cells [152]. Neural stemness contributes to and is required for both tumorigenic and differentiation potentials of tumorigenic cells. Embryonic pluripotent stem cells and induced pluripotent stem cells have been well characterized for their tumorigenicity and pluripotency, and cancer cells are well known for their tumorigenicity. However, increasing data showed that neural stem cells are both pluripotent and tumorigenic, and cancer cells are characteristic of neural stemness and display pluripotent differentiation potential [152, 158, 160, 161, 194–206]. Moreover, loss of pro-differentiation genes leads to acquirement of tumorigenicity and neural stemness in differentiated or tissue stem cells [160, 207, 208]. A latest study using genetic mouse models demonstrated that metaplastic tuft cells turn into neural-like progenitor cells in the progression of pancreatic cancer [209]. Vice versa, loss of neural stemness in cancer cells and neural stem cells via differentiation leads to the loss of both tumorigenicity and pluripotency [152, 158, 161, 210]. It may be argued why neural stemness but not the stemness of embryonic pluripotent cells plays the key role in tumorigenicity and pluripotency. The uniqueness of neural stemness is reflected by that (1) neural genes are the most conserved genes during evolution as compared with non-neural genes since founders of most neural genes have emerged during the transition from unicellularity to multicellularity; (2) the last common unicellular ancestor of metazoans is biased towards a neural state because of over-representation of founders of neural genes in the genome of *Monosiga brevicollis*, the closest unicellular relative of metazoans; (3) genes for basic functional machineries or developmental programs, such as cell cycle, ribosome, spliceosome, epigenetic modifications, are mostly enriched in embryonic neural cells; (4) as compared with non-neural genes, neural genes are characteristic of over-representation of longer genes with more exons and introns, which can generate more splicing variants and serve as more flexible scaffolds for gene regulation required for differentiation. Contrary to the unknown mesenchymal state and its regulatory networks, the property of neural stem cells is well characterized, and its regulatory networks are composed of more than 5,000 genes that are specific to or enriched in embryonic neural cells [156, 160]. These features together define neural stemness as a pluripotent and highly proliferative state upon which other cell types are derived [156, 158, 160]. This notion is reinforced by that pluripotency has a unicellular origin [211] and the default fate of embryonic pluripotent cells is neural stem cells, i.e., the “neural default model” of embryonic pluripotent cells [204, 212–215]. It is further supported that the pluripotency-like signature is maintained in the ectoderm that gives rise to neural plate, and later becomes restricted to neural crest [216]. The critical importance of neural stemness in contribution to pluripotency and tumorigenicity was systematically reviewed [155–157].

EMT contributing to cancer is mainly evidenced by the correlation between expression of EMT factors in cancer cells and cancer progression, regulation of different features of cancer cells by EMT factors, and regulation of EMT factors by others during cancer progression [14, 16, 17]. It is believed that EMT confers stemness to cancer cells but without knowing the concrete mechanisms behind [117, 217]. A few studies showed the clue that stemness factors SOX2, BMI1, OCT4, or SOX9 can be regulated by ZEB1, SNAI1, or SNAI2, thereby promoting not only stemness and metastasis of cancer cells, but also resistance to radio- and chemotherapy [218–221]. Interestingly, Sox2, Sox9, Bmi1 and Oct4 gene expression is localized to embryonic neural cells during vertebrate embryogenesis [156] (Fig. 2). Genes promoting cell proliferation, such as *CDK1* and *PLK1*, promote or are required for EMT in cancer cells [222–224]. Their expression is enriched in embryonic neural cells (Fig. 2). The pro-survival protein BIRC5 and MCL1, whose genes are enriched in embryonic neural cells (Fig. 2), were shown to regulate EMT in liver and gastric cancer cells [225, 226]. Cancer cells are characteristic of upregulated expression of epigenetic modification factors, such as LSD1 and EZH2. They are not only involved in EMT, but also regulators of immune evasion, immunotherapy resistance and stemness of cancer cells [227–231]. Genes of most epigenetic factors show enriched expression in embryonic neural cells [156] (Fig. 2). One major mechanism underlying EMT associated chemoresistance is that EMT factors are able to induce transcription of genes encoding ABC transporters, such as ABCC4 [228, 232], which is localized to the midbrain-hindbrain region of mouse embryo (Fig. 2). PI3K/AKT pathway plays essential roles in regulating EMT-TFs [233] and cancer metabolism [234]. PKM2 is involved in the regulation of aerobic glycolysis in cancer. Stimulation of EMT results in the nuclear translocation of PKM2 in colon cancer cells, which is pivotal in promoting EMT [235]. Genes of Pkm2 and Akt1 exhibit enriched expression in embryonic neural cells (Fig. 2). Chromosomal instability is a hallmark of cancer. EMT is associated with chromosomal instability [236] and the EMT transcription factor TWIST1 induces chromosomal instability and the expression of the DNA damage marker H2AX in cancer cells [237]. EMT was introduced to cancer research because it might explain cancer metastasis. Src/FAK signaling plays a central role in cancer cell migration via regulating EMT [238]. Accordingly, the genes for H2AX, Src and FAK are enriched in embryonic neural cells (Fig. 2). All the information indicates that neural stemness and its regulatory networks are responsible for different features of cancer cells. EMT factors are a few components of neural regulatory networks, it is rather rational that different components may regulate each other in cancer cells. EMT appearing almighty in the regulation of cancer cell features is merely a fiction by assigning mistakenly the roles of neural stemness to the mythical mesenchymal state.

### EMT effect in neural crest formation: misattribution of the intrinsic property of neural crest cells to mesenchymal state

Looking back on the EMT effect during neural crest development reveals the same. Locating between neural plate and epidermal ectoderm, neural crest is induced by interactions between neural plate and adjacent tissues. Neural crest cells are migratory, pluripotent and share regulatory network with cleavage stage embryos, differentiating into peripheral nervous system and many types of non-neural tissues/cells, such as melanocytes, skeletal and connective tissues, and medulla cells of the adrenal gland, etc. [163–167]. The neuroepithelial or neural plate cells are primitive neural stem cells, which are pluripotent and tumorigenic. Once committed to neuronal differentiation, they delaminate and migrate away to form the central nervous system. The property of neural crest cells is ultimately derived from neural plate cells. The typical EMT factors or markers, such as Snai1/2, Twist1, Zeb2, Sox9/10, N-cadherin, Vimentin, etc., are specifically expressed or at least enriched in either neural plate or in neural crest [157] (Fig. 2). This means clearly that migratory behavior of neural crest cells is their intrinsic property. It is really weird that the property of neural crest cells must be explained by the unknown mesenchymal state with the help of genes specific to or enriched in neural crest [239–241].

## The confusing EMT-MET cycles in developmental process and cancer progression

The mesenchyme and epithelium are considered as the basic cell types that constitute the metazoan embryos [89]. Therefore, the developmental process and cancer progression are explained by the EMT-MET cycle. During embryonic development, it is believed that MET operates as early as the 8-cell mouse embryo to form epithelial trophectoderm. In gastrula, EMT drives mesoderm formation. Both EMT and MET are employed during development of definitive embryonic endoderm, which give rise to the gut and internal epithelia of pancreas, liver, and associated glands [11, 79]. This binary classification of mesenchyme and epithelium and transitions between them mess up the process of progressive differentiation during embryogenesis, which give rise to the large diversity of cell types with specific cellular properties and physiological functions. The EMT-MET cycle describes the normal developmental process, which is generally a unidirectional process of differentiation, as a closed circle formed by adhesive and non-adhesive status, as delineated recently [249]. It is hard to understand why the change in cell adhesiveness and shape can drive the change in cellular properties including differentiation status and tissue-specific functions throughout the whole developmental process. It should be more plausible that the change is a consequence but not the cause of differentiation because differentiation needs inducing signals from other cells.

EMT symbolized by expression of EMT factors during cancer progression has been widely reported. Problems occur when E-cadherin-expressing cells are present at a metastatic site. In the context of EMT, why tumor cells sustain the expression of E-cadherin at a metastatic site remains unclear [11]. MET is an explanation of choice. This raises the same question as in development, in that cells in a tumor are classified as just epithelial and mesenchymal, and intermediate states between fully epithelial and mesenchymal states. This EMT-MET cycle does not consider the fact that cancer (tumorigenic) cells exhibit stemness and can differentiate. As mentioned above, the core property of cancer cells is neural stemness, which determines tumorigenicity and pluripotency. It was thus proposed that tumorigenesis represents the process of progressive loss of original cell identity and acquirement of neural stemness, thereby acquiring tumorigenicity and pluripotent differentiation potential [155–157]. This reminds of embryonic neural induction, a process during which ectodermal cells during gastrulation lose their epidermis fate and gain the fate of neuroectoderm, thereby acquiring pluripotency (and tumorigenicity). It further gives rise to the nervous system and other non-neural cells that are essential for the establishment of body axis. Failure of neural induction leads to failure of body axis formation, and ectopic neural induction during gastrulation causes the formation of a secondary body axis, i.e., a conjoined twin. Tumorigenic cells, including embryonic stem cells, neural stem cells, and cancer cells, exhibit pluripotency and differentiate into normal cells under instruction of embryonic inducing signals and integrate into embryonic development, contributing to formation of chimeric embryos; they cannot differentiate into normal adult tissue/organ cells and integrate into tissues/organs because of lacking of inducing signals and thus form tumors in the environment of a postnatal animal. The mutually exchangeable property of pluripotency and tumorigenicity in embryonic and postnatal stages of animals and human, and the commonality of neural induction during embryogenesis and the neural induction-like process during tumorigenesis suggest that tumors are severely degenerated conjoined twin-like structures formed in postnatal animals and human [157]. In fact, it has been well documented that different types of cells and expression of different tissue markers are detected in different tumors, including the epithelial and mesenchymal cells and their markers. The so-called tumor phenotypic heterogeneity is at least partially the result of differentiation of cancer cells, either at the primary or metastatic site [156, 157, and references therein]. From historic view, it was a type of cancer cells, the teratocarcinoma cells, that enlightened the study on pluripotency [250]. But the pluripotent property of cancer cells in contributing to phenotypic heterogeneity has been rarely considered in cancer research. Two studies at the beginning stage of EMT research proposed that epithelial and mesenchymal cells within a tumor are not generated from EMT but from cancer stem cell differentiation [119, 120]. Unfortunately, the insightful idea was not considered by mainstream studies and faded into oblivion over time. In summary, like that EMT-MET cycle cannot be helpful for understanding embryogenesis, it cannot help to understand cancer progression.

## Conclusions and perspective

After more than half a century of EMT research, it is unfortunate to find that there is almost no basis on which the EMT can be established as a scientifically meaningful concept or a general rule contributing to developmental and pathological processes. First, epithelial and mesenchymal cells being classified as two cell types is not appropriate. In general, cells within a type exhibit similar structure, function and regulatory networks that are distinct from cells in other types [251, 252]. However, epithelial and mesenchymal cells are defined according to their shapes and adhesiveness only, and both include many different cell types from embryonic stage to adulthood. It is difficult to generalize their cell state/property from the heterogeneity in epithelial and mesenchymal cells, and find suitable markers or the core regulatory networks to distinguish these cells from other cell types ambiguously. Second, cells are generally labeled as epithelial and mesenchymal from embryos to adults, and then EMT/MET are considered as a universal dogma dictating development and pathology. This is a circular, self-fulfilling argument. Third, no evidence confirms that EMT and MET could function as driving forces to promote embryogenesis and tumorigenesis. By contrast, the change in cell shape and adhesiveness should be the consequence rather than the cause of developmental process and cancer progression. Fourth, EMT is interpreted as a transition from stationary to migratory state. However, there is no clear-cut distinction in the migratory feature of epithelial and mesenchymal cells. Fifth, cells of a particular type exhibit features like shape, adhesiveness, mobility, and physiological functions. They are coupled together and defined by cell type-specific regulatory networks. Therefore, interpretation of change in cell property or state solely by the change in shape and adhesiveness is a sheer bias. Sixth, EMT cannot be described in a molecular way because of lack of reliable and universal EMT markers or factors. The history of EMT research raises the concern whether the gene-centric or cell-centric way is better for understanding developmental and cancer biology. The former has achieved great successes, but also failed in numerous cases. A cell state/property is determined by concerted co-regulation of many genes, and individual genes may not directly reflect or determine cellular phenotypes and functions. Therefore, a cell-centric view might be a better choice for understanding life and pathological processes. Literally, EMT sounds like a cell-centric concept. But in most cases, it uses a gene-centric way to answer questions in development and pathology.

The core EMT-TFs reveal actually the critical importance of neural stemness rather than the mesenchymal state in determination of cell properties. The privilege of neural stemness is predestined by the evolutionary advantage of neural genes and neural state. In contrast to the unknown mesenchymal state, the property of neural stem cells and regulatory networks of neural stemness has been largely characterized. The EMT effect during neural crest formation and cancer progression is a wrong attribution of the role of neural stemness to mesenchymal state. Moreover, the importance of neural stemness in determining pluripotency and tumorigenicity suggests that studies on developmental and cancer biology might benefit more from the research focus on neural stemness. It is time to face the contradictions and irrationality in EMT and its related concepts, reassess their value as general rules dictating developmental biology and pathology as shown in literatures, and reassess their value as research subjects if considering that pursuit of truth is still the core of scientific study.

## Data Availability

Not applicable.
